# Fractures in Parkinson’s Disease: Pathophysiology, Prevention, and Orthopedic Outcomes

**DOI:** 10.7759/cureus.92946

**Published:** 2025-09-22

**Authors:** Ali Osman, Tala Maya, Rayyan Bhutta, Natasha Doshi, Maryam Khan, Sara Shah, Paslene Periceles, Janae Rasmussen

**Affiliations:** 1 Orthopedic Surgery, Ohio University Heritage College of Osteopathic Medicine, Dublin, USA; 2 Medical School, University of Louisville School of Medicine, Louisville, USA; 3 Anesthesiology, OhioHealth Doctors Hospital, Columbus, USA; 4 Anesthesiology, Ohio University Heritage College of Osteopathic Medicine, Dublin, USA; 5 College of Medicine, Lake Erie College of Osteopathic Medicine, Bradenton, USA; 6 Orthopedic Surgery, Lake Erie College of Osteopathic Medicine, Erie, USA; 7 Orthopedic Surgery, Indiana University School of Medicine, Indianapolis, USA; 8 Orthopedics, University of Medicine and Health Sciences, Basseterre, KNA; 9 Orthopedic Surgery, Valley Consortium for Medical Education, Modesto, USA

**Keywords:** bone fragility, bone mineral density, fall prevention, fracture risk, osteoporosis, parkinson’s disease, sarcopenia

## Abstract

Fractures are an important comorbidity in Parkinson’s disease (PD), which can result in disability, mortality, and high healthcare costs. Impaired mobility, postural instability, bradykinesia, muscle rigidity, and decreased bone mineral density (BMD) contribute to the increased risk of falls, which often result in hip and upper extremity fractures. Hip fractures in patients with PD are associated with an increased hospital length of stay and a decline in functional independence. Multimodal fall prevention in PD is well established, but prevention of fractures is often underutilized, despite evidence-based interventions. Information on fall-related fracture outcomes after exercise interventions is an area warranting further investigation. Several new strategies, such as home-based telerehabilitation (TR) and pharmacologic agents, show promise but are not yet widely implemented in care. While screening tools and risk stratification methods are available, most patients with PD are not screened for skeletal fragility or entered into a structured prevention program. This review proposes a reframing of skeletal fragility in PD from a problem of secondary complications, amenable to treatment only after the fact, to a primary and expected consequence of neuromotor dysfunction in this disease. When fracture risk is considered a core and predictable outcome of PD progression, the integration of new models of care may be accelerated that combine movement therapy, bone-protective interventions, and proactive surgical planning to improve long-term outcomes.

## Introduction and background

Patients with Parkinson’s disease (PD) have an incidence of hip fracture two to four times higher and upper extremity fracture rates up to eightfold greater than in age-matched control populations [1-3]. Neuromotor features, such as bradykinesia, postural instability, and axial rigidity, all contribute to the loss of protective reflexes and automaticity in the execution of gait sequences, with resultant falls [4,5]. Additional factors, such as PD-associated sarcopenia and physical deconditioning, can be viewed as synergistic causes of biomechanical states with an increased risk of skeletal trauma [5]. A recent systematic review and meta-analysis identified more than a threefold increased risk of hip fractures in patients with PD [6,2]. Upper extremity fractures of the distal radius and humerus are also common [3]. Anterior collapse following a failed postural correction mechanism is thought to be the primary injury mechanism for both distal radius and proximal humerus fractures in PD [6].

The selective loss of dopaminergic neurons in the substantia nigra pars compacta is a hallmark of PD pathogenesis, with approximately 70% of neurons destroyed by the time motor symptoms first appear [7]. This degeneration contributes not only to motor dysfunction but also to a broad spectrum of non-motor symptoms, including sensory impairment, cognitive dysfunction, and hallucinosis, which further increase fall risk and impair post-fracture recovery [8].

Patients with PD typically have more complications after sustaining a fracture, such as increased hospital length of stay. Rarely do patients return to their pre-fracture functional baseline. Contributing factors include delayed mobilization, increased postoperative complications, and worsening of motor symptoms. In a retrospective series, patients with PD undergoing surgery had a higher rate of contralateral hip fracture and less time to ambulation compared to controls without PD [7]. The risk of other postoperative complications, such as infection, delayed bone healing, and hardware failure, also appears to be higher in patients with PD after upper extremity fracture surgery [8,9].

Yet, despite the clear contribution of PD-related neuromotor deficits to fracture risk, the current care paradigm is almost entirely focused on the prevention of falls, with limited literature focused on the management of fractures in patients with PD. Exercise-based interventions for balance impairment and fall risk have been extensively studied in PD and are considered a mainstay of fall risk reduction, but few trials report fracture as a secondary outcome [3,6]. In addition, fracture prevention care processes, such as screening for low bone mineral density (BMD), monitoring for vitamin D deficiency, calcium supplementation, or pharmacologic interventions to reduce osteopenia and osteoporosis, are not generally considered part of standard neurologic or rehabilitative care [1]. In one population-based cohort study, less than 10% of patients with PD were screened for osteoporosis after fracture [10].

This discrepancy between fracture risk and actual care is a symptom of a common perception that skeletal fragility is a secondary problem in the context of neuromotor deficits. It is becoming increasingly clear that fracture risk is a consequence of neuromotor dysfunction in PD [5]. Immobility and physical deconditioning are both recognized risk factors for fractures independent of PD, which are largely due to the development of osteopenia, osteoporosis, and sarcopenia [11]. Asymmetrical loading patterns and recurrent trauma resulting from chronic falls further increase the risk of fractures [5]. Bone loss, osteopenia, and osteoporosis, with subsequent fractures, are likely sequelae of PD due to the neurologic and biomechanical environment created by this disease. In this review, we will examine the pathophysiologic mechanisms that underlie the relationship between PD and fractures. In addition, we will highlight opportunities to improve the identification and management of patients at risk for fracture with PD.

## Review

Pathophysiology of bone loss in Parkinson’s disease

Decreased weight bearing and more sedentary lifestyles are a prominent cause of frail bones in PD. Decreased ambulatory time and repetitive dynamic joint loading are both decreased in patients with PD due to the classic Parkinsonian features of bradykinesia and rigidity. This lack of bone-loading stimulus leads to loss of BMD [12]. A reduction in dynamic joint loading, in conjunction with an overall shuffling gait pattern and diminished axial rotation, causes a failure to produce normal osteogenic mechanical strain [13]. This combination of effects leads to localized bone loss, described as osteopenia and osteoporosis, in immobilized segments, as well as more generalized skeletal demineralization that can be detected by measuring reduced BMD at multiple skeletal sites, such as the distal radius and femoral neck [14]. This, in conjunction with a notably high fall and fracture risk in individuals with PD, has contributed to the increased interest in bone health in this population [14,15]. Notably, patients with PD experience hip fractures at rates two to four times higher and upper extremity fractures up to eight times higher than age-matched controls [1-3]. Targeting improved mobility earlier in PD may help improve walking patterns, which may help to reduce the risk of osteopenia and osteoporosis.

Neuroendocrine imbalance is a related process that has been linked to bone fragility in PD. The loss of dopaminergic neurons in the brain and downstream effects on the hypothalamic-pituitary-gonadal axis lead to reductions in protective sex hormones, including estrogen and testosterone, both of which have key roles in bone formation and remodeling [16]. In addition, vitamin D deficiency is highly prevalent in the PD population [17,18]. Serum 25-hydroxyvitamin D levels have been demonstrated to be significantly lower in patients with PD compared to age-matched controls [17,18]. Insufficient vitamin D results in multiple processes that promote decreased BMD, as it leads to an inability to absorb calcium and mineralize the bone matrix, which can cause sarcopenia (age-related, progressive muscle weakness). Evidence suggests that vitamin D abnormality occurs in people with PD early in the disease process, as abnormal vitamin D levels were observed even at Hoehn & Yahr stages I-II [17]. The Hoehn & Yahr scale is a commonly used tool for assessing Parkinson’s disease progression, with stage I indicating unilateral involvement and minimal or no functional disability, and stage II indicating bilateral involvement without balance impairment. Proactive evaluation of neuroendocrine function and treatment with vitamin D supplementation may be one way to combat the effects of low BMD in people with PD.

Chronic inflammation is another cause of increased bone fragility in PD, acting through mechanisms that increase osteoclast activity. Chronic elevation of circulating interleukin-6 (IL-6) and tumor necrosis factor-alpha (TNF-α) has been described in PD [19,20]. Both cytokines are recognized in enhancing osteoclast differentiation and function [19,20]. In addition, genetic risk factors, such as an LRRK2 mutation, can increase microglial activity and peripheral immune activation, potentially further increasing the systemic release of inflammatory cytokines [21]. The increased release of cytokines can negatively impact the structure and strength of bone [21]. A recent meta-analysis of inflammatory biomarkers in PD showed significantly elevated IL-6 and TNF-α in the serum and cerebrospinal fluid of PD patients, with greater levels correlating with more severe disease and markers of bone turnover [20]. This state of chronic inflammation works in conjunction with the lack of mechanical activity, which increases the risk of bone loss. Ultimately, weakened bone (osteopenia and osteoporosis) increases fracture risk susceptibility and impairs fracture healing after orthopedic trauma. Anti-inflammatory therapies and monitoring of these inflammatory biomarkers have the potential to reduce bone fragility in the PD population.

Sarcopenia, or loss of muscle and strength, is a final process related to bone fragility in PD. Sarcopenia is the progressive and generalized loss of skeletal muscle mass and function, which is classically described through the aging process [22]. However, it is highly prevalent in individuals with PD even early in the disease course [22]. Muscle activity and contractions generate the primary mechanical stimulus necessary for bone maintenance, so continued decreased muscle utilization leads to suboptimal bone remodeling and adaptation. Sarcopenia subsequently leads to a higher risk of falls and fractures in patients with PD. Many factors likely contribute to sarcopenia in the context of PD, including physical inactivity, nutritional deficiencies, and neuromuscular degeneration. This further diminishes muscle-bone coupling and, by extension, structural support, especially of the weight-bearing bones of the lower extremities. As such, sarcopenia in the setting of PD is associated with an increased risk of hip fractures and may partly explain the two- to four-fold higher incidence of hip fractures reported in individuals with PD compared to age-matched controls [1-3,23]. In addition, the coexistence of sarcopenia and osteoporosis, referred to as osteosarcopenia, has been associated with a high comorbidity burden and unfavorable rehabilitation outcome after a fracture in both patients with and without PD [24]. Early introduction of muscle-preserving strategies, such as resistance exercise and nutritional support, will likely be an important tool against bone weakness for patients with PD.

Clinical manifestations and diagnostic challenges

Skeletal fragility is an often-overlooked but clinically significant problem linked to PD, and it can often go unreported until a fracture occurs [25]. Underdiagnosis may be due to bone loss in this population, typically occurring without clear clinical markers or warning signals. From a diagnostic and functional standpoint, the problem is critical, since fragility fractures, typically from low-energy mechanisms like a ground-level fall, frequently serve as the first indication of compromised bone health.

The hip and upper extremities are where fractures in PD are often noted, with proximal humerus and distal radius fractures being common in this patient population [26,27]. The distinct biomechanics of falls in PD, which usually result from anterior collapse, are often attributed to failed postural corrections and impaired balance. Postural instability, bradykinesia, axial stiffness, and diminished protective reflexes are some of the neuromotor characteristics of PD that lead to an elevated frequency of falls, notably forward falls, which ultimately increase the risk of fractures [28,29]. PD-related gait abnormalities, which impair the normal loading mechanics and balance during movement, exacerbate this condition.

Although generally systemic, the reduction of BMD in PD may be more noticeable in the upper limbs, such as the distal radius, based on some findings [30]. This may reflect a combination of diminished muscle mass, less mechanical loading, and utilizing the wrist to help brace during a fall. This fragility is made worse by sarcopenia and physical degeneration, which are common in PD patients due to decreased mobility and physical activity [31,32]. These circumstances impede the mechanical support system for bones. These musculoskeletal changes play a key role in the progressive biomechanical decline seen with PD. Studies utilizing the Unified Parkinson's Disease Rating Scale (UPDRS) showed a strong relationship between the level of motor impairment and fracture risk [33]. Higher UPDRS scores have been linked with worse outcomes after fractures and a higher likelihood of frequent falls [33]. Skeletal injury can be reliably predicted by the level of motor dysfunction, particularly loss of balance and control while walking.

In clinical practice, standardized bone health screening is generally lacking, including in patients with PD. Even after a fracture, dual-energy X-ray absorptiometry (DEXA) scans, the most accurate method for determining the density of bones, are seldom carried out in individuals with PD [34]. A substantial care gap was observed in one cohort study, where less than 10% of PD patients received an osteoporosis screening following a fracture [35]. More advanced methods, like high-resolution peripheral quantitative computed tomography (HR-pQCT), are even less utilized [36]. These methods can reveal details about bone microarchitecture but are rarely used outside of the research setting. There are currently no general or disease-specific guidelines requiring or recommending routine bone health monitoring for PD patients [37]. Due to this, individuals with PD may never have their bone fragility evaluated or engage in formal preventative programs. Pharmaceutical treatments for fracture prevention, supplements (such as calcium and vitamin D), and routine bone density testing are frequently left out of standard neurologic and rehabilitative treatment routes.

This underdiagnosis can be attributed to the lack of awareness that bone health is a primary concern in the care of PD [38]. In reality, one of the primary, predictable, and mechanistically linked effects of neuromotor deterioration is increased fracture risk. Osteopenia and osteoporosis can be attributed to inflammatory causes, muscular atrophy, persistent asymmetrical loading, and recurrent injury from falls.

In light of these findings, it is vital that fracture risk be reconsidered as an essential aspect of PD advancement, which warrants improved attention, such as fall prevention. Ongoing examinations of BMD, early detection of risks, and interdisciplinary management techniques, including individualized exercise regimens and bone-protective medication, should be included in PD standard of care [39,40].

Interventions and management strategies 

In PD, early intervention and management strategies should be employed to reduce the risk of fracture, as loss of dopaminergic neurons in nigrostriatal circuits can lead to downstream effects of motor impairment. Subtle issues with gait and balance in the earlier stages of PD, along with a significant risk of falls, support a need to integrate physical therapy into the care plan of PD patients [41]. Organized exercise and physical therapy can allow for enhanced function of motor and non-motor symptoms [41]. Early fall assessments and prevention are of critical importance, as supported by one study that notes almost 80% of recently diagnosed PD patients experience falls within 4.5 years [41]. A quarter of these patients expressed experiencing falls prior to diagnosis [41].

Weight-bearing exercise can aid participants by increasing bone density in the femoral neck region and suppressing sclerostin production [42]. This may be of special importance, as the therapeutic benefits of levodopa, a commonly used medication for PD, may be coupled with inhibition of osteoblast activity, which may modify the extent to which mineralization occurs [42].

Beyond physical therapy and exercise, pharmacologic interventions also play a critical role in reducing fracture risk in PD. Bisphosphonates can serve as a useful pharmaceutical tool to strengthen bone in those with low BMD [43]. The Trial of Parkinson’s and Zoledronic acid (TOPAZ) study is collecting information on the use of zoledronic acid in lowering fractures [37].

Removing tripping hazards and improving lighting, along with an array of other home safety modifications, can reduce fall risk [44]. Disease severity and deficits specific to the patient can be considered upon evaluation of fall risk.

Bone health is a factor that may be overlooked during neurological care of those with PD. In one study, 73% of patients did not have an up-to-date fracture risk assessment [45]. Caregivers and those around patients can also be educated on transfer techniques, mobility improvement strategies, and integration of exercise routines. Furthermore, risk factors should be assessed and medications should be checked to reduce fall risks [44]. Follow-up visits can be done to ensure that bone health is not compromised [46]. PD itself has emerged as a condition requiring assessment of fracture risk [45]. Emerging models involving telehealth have led to accessible training on fall prevention and bone health maintenance [47]. Regular screenings and early interventions can improve the quality of life for PD patients.

Emerging and adjunctive strategies 

Telerehabilitation (TR) can be defined as the application of digital technology in the delivery of therapy and interventions, which are designed to promote and improve an individual’s health, while also allowing healthcare providers to monitor their patients’ progress and safety remotely [48]. This makes TR applicable for patients with PD for a variety of reasons. For example, transportation to and from a healthcare provider can be a substantial issue for PD patients, who may be burdened with mobility problems, fatigue, or motor fluctuations. Thus, the possibility of receiving rehabilitation care at home represents an advantage that lessens both physical and logistical burdens for these patients.

A small study of 15 patients with mild-to-moderate PD was performed at a movement disorders clinic [49]. The participants received 10 weeks of TR consisting of twice-weekly physical and occupational therapy [49]. The patients had significant and sustained improvements in their physical and occupational therapy goals, demonstrated high adherence to home-modification recommendations, and had no adverse events or dropouts during the telemedicine program [49]. These results suggest that telehealth may provide expanded access to specialized care for patients with mobility limitations or significant distances from movement disorder clinics. The TR, having high compliance with no dropouts, indicates its effectiveness is at par with in-person rehab. This is particularly advantageous for PD patients living in rural regions and those unable to bear the costs associated with long-distance travel. Implementing TR reduces the risk of falls during transit to a clinician's office, as PD patients can now receive treatment and continue to make progress in the comfort of their own homes. Insurance barriers and the reliance of many patients on caregivers often limit access to rehabilitation services and make travel to specialized clinics burdensome. Telehealth can help overcome these challenges by offering accessible, home-based care that reduces financial strain.

A study involving adults over the age of 65 revealed that the integration of telehealth with exercise programs and smart home technologies significantly diminished the risk of falls while enhancing balance and fall efficacy among older individuals [50]. The research further suggests that future investigations should concentrate on leveraging smart home technology and artificial intelligence to forecast falls [50]. Older adults represent the demographic most susceptible to developing PD, rendering this study particularly relevant for those afflicted with this condition. Given that the combination of telehealth, exercise programs, and smart home systems can reduce fall risk and enhance balance and confidence in geriatric patients, analogous interventions could assist patients with PD in achieving better neuromuscular control and safer living environments.

Wearable systems that utilize sensors and insoles are prevalent and dependable technologies for assessing fall risk and providing real-time monitoring [51]. Although there remain ongoing challenges and opportunities for enhancement to create effective, cost-efficient devices [51], this category of wearable technology has facilitated the development of efficient fall detection systems aimed at improving safety for the elderly. By employing accelerometer and gyroscope sensors, these systems have demonstrated remarkable accuracy, achieving sensitivity and specificity rates exceeding 97% and 99%, respectively, based on experimental data from numerous participants [52]. Patients with PD often encounter a sudden loss of balance that increases their fall risk, frequently occurring unexpectedly and without immediate assistance available. A wearable fall detection system can deliver timely alerts with accurate Global Positioning System (GPS) locations, ensuring prompt assistance from caregivers for individuals with PD. Facilitating faster emergency responses and ongoing monitoring, it can contribute to minimizing the severity of injuries and improving overall safety for patients with PD.

Various medications can diminish the chances of falls in patients suffering from PD. Denosumab enhances BMD, and teriparatide, an osteoanabolic agent, facilitates the creation of new bone [53]. The capacity of denosumab to consistently improve BMD presents a promising preventive approach by increasing bone resistance to fractures over time. In this regard, this is particularly important for patients with PD who have an increased risk of falls. Prolonged denosumab therapy may, therefore, create a more robust skeletal framework and contribute to lessening the severity of fractures. Teriparatide has a reconstructive effect on bone microarchitecture that is degenerated due to age, disease, and physical inactivity. Research is currently being conducted to examine the potential benefits of using denosumab in combination with high doses of teriparatide [54]. Results have shown this combination to result in significant increases in bone mass, density, and estimated strength in key areas of the hip and spine, outperforming taking the drugs alone [54].

In a study that looked at the effectiveness of these treatments on premenopausal women with idiopathic osteoporosis, a 24-month teriparatide treatment significantly increased BMD at the spine and hip and improved bone microarchitecture [55]. This was then followed by 24-month denosumab treatment, which led to a significant increase in the previous improvements made by teriparatide, resulting in an overall significant increase in bone strength as measured by a DEXA scan [55]. The sequential strategy of these treatments appears to be successful in improving bone health. The synergistic effects between denosumab and high-dose teriparatide were shown to significantly increase bone density and bone strength in areas commonly prone to fractures, including the hip and spine. A similar level of benefit may be seen when this combination is given to patients with PD. Improvement of bone strength in these patients would greatly contribute to decreased fracture risk as a result of falling and subsequent sequelae, such as loss of independence and fractures. The quality of life of patients with PD would be greatly improved as a result of improved pharmacologic interventions for increasing bone health.

Diet modification can also help to further decrease fall risk among PD patients. In a study following older Spanish adults over a 3.5-year period, 19.4% of participants experienced one or more falls [56]. Study participants who followed the Mediterranean diet closely, primarily consisting of fish and seafood, legumes, whole grains, and vegetables, had a 28% lower risk of falling than those with the lowest adherence [56]. Consuming two or more daily servings of vegetables was strongly associated with a lower risk of falling [56]. Overall, the Mediterranean diet’s ability to prevent falls is likely to be a result of the cumulative impact of many different foods, such as the decrease of blood lipids and inflammatory and oxidative stress markers, and the improvement of insulin sensitivity, endothelial function, and its antithrombotic function [57]. Mediterranean diets are known to provide essential vitamins and antioxidants, which can help improve motor function and coordination, and a high-protein diet and implementation of omega-3 fatty acids in the diet can also lower the risk of falls [58].

Dietary protein intake and clinical data from 807 older adults in the Framingham Original Cohort Study were analyzed to assess the relationship between protein consumption, weight changes, and self-reported falls over time [54]. Higher dietary protein intakes were associated with reduced odds of falling, likely due to the relationship between protein source and lean muscle mass. Similarly, a randomized controlled trial involving 2,157 adults aged 70 and older across five European countries tested the effects of omega-3 fatty acids on fall incidence over three years, with fall rates recorded prospectively and analyzed using a modified intent-to-treat approach [59]. This study found that there was a 10% decrease in falls after implementing omega-3s in the diet [59]. By reducing inflammation and enhancing muscle mass, PD patients can reduce their risk of falls by implementing simple nutrition changes into their diet.

Neuromuscular electrical stimulation (NMES) is the repeated application of current to produce contraction of innervated muscle by depolarizing local motor nerves [60]. Studies show that this technique can help those with critical illnesses, such as PD. NMES can be a valuable tool by strengthening key muscle groups without requiring active effort from the patient, which can be difficult due to their impaired motor control. Improved stability, posture, and mobility can decrease the risk of these patients falling. A randomized study of 25 patients experiencing their first acute anterior cruciate ligament (ACL) tear explored the effects of NMES on the quadriceps [61]. NMES treatment helped to lessen the atrophy in fast-twitch fibers and maintained contractile performance in slow-twitch fibers [61]. By strengthening these muscle fibers and improving their function, NMES might enhance muscle responsiveness and balance control in patients with PD, potentially reducing their risk of falls. Additionally, NMES has been utilized to increase lower limb venous return, reducing thrombotic risks [62]. This is particularly important for patients with PD who struggle with maintaining physical mobility and are at a higher risk of developing a thrombus (blood clot). 

Risk stratification and screening tools

Risk stratification in patients with PD reveals several tools with the potential for integration into common clinical practice. The Fracture Risk Assessment (FRAX) tool estimates the 10-year risk of hip and other major osteoporotic fractures, which can be utilized both with and without BMD data [63]. This is of distinct importance for PD patient populations, who are oftentimes limited in terms of undergoing DEXA scans to assess bone density due to factors including patient frailty [63,64]. However, FRAX is not routinely used in practice for PD [63-66]. Several audits and cohort studies illustrate that only a small subset of PD patients receive FRAX-related assessments, and osteoprotective measures are seldom implemented or cross-coordinated by providers in neurology and rehabilitation sciences [63-66]. Even at the community level, extensive studies demonstrate that systematic screening is uncommon, resulting in widespread underestimation of bone health needs in patients with PD [67].

Key risk factors for osteoporosis and fracture in PD are well-established and should inform screening strategies. Female sex is regularly associated with an elevated risk of fracture, along with low body mass index (BMI), which amplifies bone fragility both in PD and general osteoporotic research groups [63,68-70]. Similarly, the usage of medications like selective serotonin reuptake inhibitors (SSRIs) and steroids (e.g., glucocorticoids and corticosteroids) also increases fracture risk through effects on bone metabolism and fall risk (e.g., through gait and stability issues) [64,66,71,72]. Additionally, sarcopenia frequently occurs as a comorbidity in PD patients, further increasing fall and osteoporotic risk, as indicated through both epidemiologic and meta-analytic studies [73-75]. These risk factors act in a synergistic way to produce a higher fracture burden in PD groups as compared to non-PD groups, underscoring the need for preventative, multifactorial risk assessments [65,75].

Routine functional assessments, including the timed up and go (TUG), gait speed, grip strength, and short physical performance battery (SPPB), are paramount in risk stratification, especially as it pertains to PD patients [73-75]. The TUG test remains the gold standard with respect to mobility and dynamic balance evaluation in PD populations [74]. TUG is advantageous since it is time-effective, correlates strongly with functional independence, and reliably distinguishes patients at high risk for falls and subsequent fractures [74]. In a similar fashion, gait speed as either an independent measurement or one of three components in the larger SPPB package proves useful both as a direct and accurate predictor of future falls as well as a means to identify underlying sarcopenia and functional decline, both common and dangerous comorbidities in PD [73,75]. Grip strength, on the other hand, has been verified as a biomarker for diagnosing sarcopenia in patients with PD. Low handgrip strength is related to higher levels of disability, frailty, and risk of falls and fractures in PD, helping to elucidate neuromuscular degeneration as a mechanism of overall mobility risk [73,75]. Although the SPPB is a less common assessment in PD-specific clinics than it is in general geriatric practice, it allows a multidimensional evaluation of lower extremity function through balance, gait, and sit-to-stand tests [74]. Studies have found it discriminates between patients at different levels of risk for poor outcomes, and it has been found to be reliable and feasible in older adults with movement disorders, although with some difficulty in validation for end-stage PD [74,64]. Of note, these functional measures augment more conventional risk calculators like FRAX by providing functional, patient-specific information about propensity to fall and musculoskeletal performance [74,73]. Integrating these tools into a standardized PD assessment enables clinicians to precisely identify those patients most vulnerable to mobility loss and serious injury and to tailor interventions accordingly, thereby taking actionable steps toward fracture prevention and optimization of overall care [75,64].

Although such validated assessment tools exist, many neurologists, orthopedic surgeons, and rehabilitation providers do not routinely implement fracture risk screenings and measures in PD patients [63,64,67]. National audits and other studies have demonstrated that bone health and osteoporotic risk are often overlooked in PD patient encounters, culminating in gaps in the realms of primary, secondary, and tertiary care models [63,64,67]. Osteoporotic therapy and relevant risk evaluations further exemplify both local and global deficits in provider awareness, provider knowledge, and systemic implementation [63,65,66,75]. A potential reason for these gaps is due to limited interdisciplinary collaboration between specialists who work with patients with PD, including neurologists, rehabilitative specialists, geriatricians, and orthopedic surgeons [63]. Current reviews, although limited in this field, echo that this underdeveloped interprofessional collaboration pairs with currently existing fracture risk algorithms that are not tailored, in general, to patients with mobility disorders [63,65,67]. These limitations discourage adoption and raise concerns about the accuracy of fracture risk tools in PD [64,65]. Consequently, the literature repeatedly calls for the integration of bone health assessment and preventive intervention as a standard component of multidisciplinary PD management to address these shortcomings and prevent avoidable fractures [63,64,66].

Special populations and complex cases 

PD is the second most common neurodegenerative disease, commonly characterized by both motor and non-motor manifestations that notably reduce quality of life [76]. These clinical symptoms, combined with secondary factors such as decreased physical activity, osteoporosis, and vitamin D deficiency, impact weight-bearing activity and are linked to an impairment of balance and mobility, predisposing patients to frequent falls. They also lead to accelerated bone loss, which increases the likelihood and severity of injury from falls [77]. Of these injuries, hip fractures, particularly intertrochanteric and femoral neck fractures, in patients with PD are reported to be most commonly seen in emergency rooms [78]. Hip fractures in patients with PD often occur within the first five-year prodromal period [79]. Furthermore, hip fractures carry the highest burden in PD, with one-year mortality rates often exceeding those observed in age-matched non-PD populations [80]. Additionally, cohort studies conducted on patients with PD and age- and sex-matched controls found a higher ten-year mortality rate among older male participants [81]. These reports suggest that the lower extremities are often more prone to injury, with hip fractures manifesting as the most severe and consequential. These injuries represent a major source of morbidity, mortality, and long-term loss of independence in patients with PD. In addition to the disease-related symptoms discussed previously, there is also a direct relationship between advanced age and structural damage. The skeletal fragility that ensues due to a low BMD, compounded with the increased likelihood of falling, results in a higher susceptibility to fragility fractures due to the effects of aging [82]. Moreover, in a study conducted by Siris et al., the authors concluded that the median survival time of patients with PD suffering from hip fractures was 31 months (95% CI, 25-37 months), as compared to 45 months (95% CI, 39-50 months) for the healthy control group (p = 0.007) [83].

Outside of the rates of mortality, the decline in the normal daily functions caused by hip fractures in patients with PD is noteworthy, as many individuals are unable to return to their independence prior to sustaining the injury. Literature suggests that among this population, the rates of institutionalization due to hip fractures are significantly higher (adjusted odds ratio (AOR) 4.06, 95% confidence interval (CI)) as compared to non-PD control patients, with many requiring residence in long-term care facilities or skilled nursing placement as a result of the difficulty in regaining ambulation prior to the injury [84]. Cross-sectional studies conducted by Safarpour et al. suggest nearly 25% of individuals with PD ultimately reside in a long-term care (LTC) facility, with women facing higher chances of placement in a nursing home. Patients with PD are also often at an increased risk of recurrent injuries that require revision surgical procedures for displaced and nondisplaced fractures compared to healthy matched controls (11% versus 4%; p = 0.005) [80]. Further contributing to the likelihood of patients with PD residing in LTC facilities, the failure of fixation rates was found to be significantly higher in PD as opposed to the control group (22% versus 5%; p = 0.01) [83]. Fractures in PD often result in delayed healing, prolonged recovery, and postponed surgical intervention. Particularly in hip fractures, these factors greatly reduce mobility, which ultimately results in a loss of functionality due to inactivity of the muscles and joints [80]. This suggests that the rehabilitation rate after sustaining a hip fracture is notably more challenging and prolonged in patients with PD due to the increased length of hospital stays and bone weakening. Given the presentation of PD, worsening of symptoms with disease progression, and the recurrence of injury due to fall risks, prevention is a critical component of disease management to ensure patient safety.

Patients suffering from advanced PD or dementia, or those residing in LTC facilities, are among the most vulnerable populations to fracture-related complications. However, they are paradoxically excluded from standard fracture and injury prevention efforts. These individuals are at a disproportionately higher risk of lower extremity fractures due to the combination of factors that lead to bone weakening, such as immobility, vitamin deficiencies, osteoporosis, and advancing motor dysfunction from disease progression [85]. The standard criteria for diagnosing the onset of osteoporosis depend on the fracture fragility, a T-score ≤ −2.5 at regions like the lumbar spine or femoral neck on DEXA exam, or a T-score between −1.0 and −2.5 with elevated fracture risk as determined by the online tool known as the FRAX [86]. BMD testing is another recommended screening tool used for diagnosis on the basis of age and risk factor status. However, there are some reported discrepancies regarding the definition of osteoporosis as opposed to osteopenia [87]. Despite the increased risk and predisposition to complications, methods such as DEXA scans, vitamin D testing, and osteoporosis pharmacotherapy are underutilized in patients with neurodegenerative disorders [88]. Therefore, patients within this demographic are underdiagnosed and receive suboptimal treatment.

Additional factors that could account for the exclusion of this demographic from prevention programs include shifts in perspective related to the disease, comorbidities, disease stage, and logistical barriers. Patients with PD ultimately develop dementia as part of the disease progression [89]. However, the time from the onset of the symptoms to the onset of dementia varies between patients [89]. For patients with advanced PD living in LTC facilities who reach the stage of dementia, the primary focus shifts from prevention to the optimization of the quality of life. Therefore, at this stage of the disease, participating in interventions is often seen as a burden rather than a productive effort [89]. In LTC facilities, the occurrence of comorbidities such as recurrent infections, chronic pain, cardiovascular instability, and orthostatic hypotension often takes precedence over fracture prevention efforts [90]. Additionally, these facilities may also face resource and staffing limitations, which may lead to a lower capacity to address the complications resulting from PD, ultimately resulting in higher rates of fractures due to falls.

Moreover, the advanced stage of PD is characterized by notable neurodegeneration and a loss of dopaminergic neurons [91]. These neurons are critical for motor movements, so this damage leads to the inability of the brain to control daily movements. Most notably, it causes the characteristic tremors and impacts the gait of patients with PD. Since this damage has already occurred when a patient reaches the advanced stages, they are deemed ineligible for enrollment in many prevention programs, since these programs often target earlier stages of the disease. Patients in LTC facilities may also often face logistical barriers, including limited access to transportation, restricted schedules, and financial burden, which can impede patient involvement in prevention programs [92]. This financial impact serves as the most commonly cited cause of patient exclusion from prevention programs due to the direct costs associated with travel and lodging or the indirect costs associated with missed days of work or additional medical testing [93]. Unfortunately, these barriers often lead to fracture-related injuries that could be easily prevented with adequate screening, supplements, and therapeutic medications.

Due to the rarity of this demographic’s enrollment in prevention programs, it is predicted that the number of institutionalized patients with PD will rise sharply in the coming decades due to the aging of the population, leading to an increase in PD prevalence and improved quality of care, leading to a prolonged survival in advanced disease stages [94]. There are simple interventions that can be used to decrease the risk of falls and further prevent hip fractures in patients with advanced PD, dementia, and those living in LTC facilities. This includes the use of bed rails, assistive devices such as canes or walkers, wearable health technology such as emergency alert medical devices, and ensuring that walkways are free of clutter [95]. Another intervention that can be used is the integration of psychosocial therapy for patients with PD who have developed dementia. This method is a group-based therapy that aims to improve cognition and quality of life in people living with dementia [96]. This suggests the incorporation of regulated schedules and regular participation in group activities. Failure to implement interventions of this nature represents several missed opportunities to prevent harm and exemplifies the essential need for system-wide attention.

As a result of the multifaceted and complex nature of PD, optimal care requires communication across several specialties to limit fragmented care resulting from inadequate coordination [97]. Since PD is a progressive disease that affects both motor and non-motor functions, it is essential that this condition be managed in a holistic manner across neurology, orthopedic surgery, geriatrics, and rehabilitation specialists. In patients without PD or related diseases, there is a balance between the activity of direct and indirect feedback pathways of the basal ganglia [97]. The direct pathway enables motor movement, whereas the indirect pathway inhibits movement. However, in PD, the direct pathway is inhibited because of a loss of dopamine D1 stimulation, while the indirect pathway is activated because of the release of dopamine D2 inhibition, both leading to reduced movement [96]. The marked involvement of neuronal cells indicates the need for neurologists to evaluate symptoms, conduct neurological exams, and confirm a diagnosis by utilizing brain imaging. Similarly, the effect of the neuronal feedback dysregulation on motor movements signifies the importance of orthopedic specialists in managing musculoskeletal symptoms that arise or are exaggerated by PD. Additionally, these specialists can utilize the previously mentioned screening methods, such as DEXA scans, FRAX scores, and BMD testing, to determine the extent of bone loss and gait abnormalities. Geriatric specialists have a key role in managing non-motor symptoms, such as cognitive decline, as well as comorbidities, such as infections, in older patients with PD. Furthermore, the role of rehabilitation specialists is important in the management of PD due to their expertise in improving mobility, strength, and balance through the use of patient-centered exercise regimens, training programs, and repetition of daily living activities in order to promote patient independence. This multidisciplinary coordination prevents oversight in patients with PD since each specialty is best suited for the many different aspects of the disease.

Future directions and recommendations

Typically, fracture risk assessments include falls as an independent factor, which excludes the approximately 60% of patients with PD who experience falls [81]. An adjusted fracture risk screening geared towards patients with PD could reduce this gap. Future directions must include studies that further analyze interventions that combine pharmacologic treatment options along with the implantation of early intervention [97]. There has been an association with increased risk of mortality related to hip fractures in patients with PD that varies in age [97]. Due to the lack of research in this area, there is a need for further research that promotes age-specific interventions in this population. Patients with PD would benefit from rehabilitation that is tailored to their needs and fracture prevention [97]. A multidisciplinary approach would enhance the prevention of fractures by expanding access to DEXA scans and home safety evaluations. Those with great fragility are usually not widely included in research; however, fragility fractures, specifically hip fractures, are directly associated with poor outcomes [97]. An integrated approach should be adopted to properly include PD as a risk factor in fracture risk assessment in the development of treatment in this vulnerable population. A dynamic and stable plan for a continuity of care post-fracture through home safety evaluation, rehabilitation, patient education, and continued fall risk evaluation can reduce the burden of fractures in patients with PD.

## Conclusions

Fractures in patients with PD are a largely preventable public health issue associated with a high burden of disease. As life expectancy for individuals with PD has increased over time, the number of new fractures and fracture-attributable morbidity, hospitalizations, re-hospitalizations, nursing home placements, and deaths is also increasing. Neuromotor decline, skeletal demineralization, and chronic inflammation lead to an inherently high-risk biomechanical environment for fractures to occur in the context of PD. In this setting, fractures are often associated with significant morbidity, with decreased function and increased LTC facility placement after a fracture. These negative outcomes have the potential to be avoided through early intervention. However, bone health and fracture risk are not commonly considered in the management of PD. The utility of early screening for bone fragility/fracture risk, which can be evaluated with DEXA, the FRAX scores, and functional measures, to identify patients at risk prior to injury, has not been consistently employed. Optimization of care is possible with tailored pharmacologic therapy, physical therapy, and home safety measures and may lead to a lower fracture burden and improved long-term outcomes. Nutritional optimization with adequate vitamin D and calcium intake, as well as diet overall, along with pharmacologic interventions such as bisphosphonates and teriparatide, should also be considered in this high-risk population as a part of a comprehensive approach to fracture prevention. If we begin to view skeletal fragility as a primary, modifiable PD outcome rather than a secondary consideration, we may be one step closer to changing clinical practice guidelines in this high-risk population. A multidisciplinary, fracture-prevention-focused approach to PD, including specialists like neurologists, rehabilitation specialists, and orthopedic surgeons, may reduce rates of fractures and preserve mobility and function for the growing PD population.
